# Straining to define a healthy microbiome

**DOI:** 10.1128/msphere.00797-24

**Published:** 2025-07-11

**Authors:** Anna M. Seekatz

**Affiliations:** 1 Department of Biological Sciences, Clemson University 170362, Clemson, South Carolina, USA; University of Michigan, Ann Arbor, Michigan, USA

**Keywords:** fecal microbiota transplantation, human microbiome, gut microbiota, gut microbiome, probiotics

## Abstract

In 2020, I wrote an mSphere of Influence commentary on two studies that shaped my research perspective on the human gut microbiome (McNulty et al., Sci Transl Med 3:106ra106, 2011, https://doi.org/10.1126/scitranslmed.3002701; Hamilton et al., Gut Microbes 4:125, 2013, https://doi.org/10.4161/gmic.23571). The microbiome field has continued to progress since the publication of these studies over 10 years ago, emerging as a considerable factor in almost all areas focused on disease development. My previous commentary highlighted two areas that piqued my interest early on in my career: (i) that the extant microbial community should be considered when proposing to manipulate the microbiota, such as via probiotics or fecal microbiota transplantation, and (ii) that realized (i.e., transcribed) functional changes of the microbiota may occur independent of changes in its composition. Since writing that commentary, two microbiota-based therapeutics for the treatment of *Clostridioides difficile* infection have been approved, highlighting the potential success of using the microbiota to treat or prevent disease. Despite these wins and ever-growing evidence of the importance of the microbiome in managing our health, translating mechanistic studies into therapeutic value has been slower. In this minireview, I expand upon two large questions that would increase our ability to translate the microbiome into therapies, highlighting both historical and recent progress.

## INTRODUCTION

The field of human microbiome science has continued to grow exponentially in the last decade. Knowledge surrounding the impact of the human microbiota, the organisms inhabiting our organ systems, on our health has surpassed associations, and mechanistic studies are published on a regular basis. The promise of using this knowledge to develop microbiome-based therapeutics has also grown; two FDA-approved live biotherapeutic products (LBPs) are now available on the market to treat recurrent infections caused by *Clostridioides difficile* (1, 2), with a third showing promise in phase 2 clinical trials (3). Other promising disease targets for LBPs include inflammatory bowel disease (4), ulcerative colitis (5), and reducing gut colonization by antibiotic-resistant organisms (6, 7). These accomplishments support a positive outlook for microbiome research, which has historically been plagued with a reputation for being too associative.

Yet, progress on advancing microbiome-based therapies beyond treating *C. difficile* has been slow. The microbiome is inherently complex, hindering scientific reproducibility and translation of mechanistic microbiome research into actual therapies. This includes some initial failures of clinical trials for treating *C. difficile* infection (CDI), dampening enthusiasm for the development of microbiome-based therapeutics (8). Despite consistent studies indicating a role of the microbiome in disease, many research areas may question the utility of microbiome research. How does one expand from associative studies into mechanism to translate these findings into therapies or preventative actions?

In this minireview, I highlight the historical perspective and recent progress on two aspects of microbiome research I feel are critical in developing effective microbial-based therapeutics:

- How does strain diversity contribute to our understanding of a “healthy” microbiome?
- How does the extant (i.e., recipient) microbial environment influence the intended function or impact of microbiome-based therapeutics?

## INCORPORATING STRAIN-LEVEL INFORMATION INTO THE EVOLVING DEFINITION OF A “HEALTHY” GUT MICROBIOME

In 2007, the Human Microbiome Project (HMP) set forth the aim of defining a healthy microbiome across multiple body sites (9). For the gut microbiota, a healthy gut can be expected to harbor high abundances of the phyla Bacteroidota (formerly known as Bacteroidetes) and Bacillota (Firmicutes), with lesser abundances of Verrucomicrobiota (Verrucomicrobia), Actinomycetota (Actinobacteria), and Pseudomonadota (Proteobacteria) (10, 11). Within each of these phyla, variability at the species level is observed across the human population. Early efforts to capture comprehensive species or strain diversity mostly relied on culture-independent sequencing methods based on the classification of the conserved 16S rRNA gene (12), which remains the most common method of identifying changes in microbiota across cohorts or time (12, 13). In initial HMP studies, the number of operational taxonomic units (OTUs) that represented different species was estimated to be in the hundreds (14). In the past 7 years, this number has been increased to the thousands based on more comprehensive strain-level diversity defined by unique 16S rRNA sequence reads (amplicon sequence variants) (15) or collating databases of metagenome-assembled genomes (MAGs) constructed from metagenomic data, which represents the collective genes of a microbiota (16–19). Most recently, Leviatan et al. constructed 3,594 reference microbial genomes from >241K MAGs (18), further expanding species and strain-level representation of the healthy gut microbiome.

These metagenomic and MAG-based estimations of microbiota diversity have expanded the number of species hypothesized to encompass a healthy microbiome, aiding our ability to associate certain species with geography, lifestyle, and environment across the “healthy” human gut microbiome. For instance, the gut microbiota of non-Westernized populations appears to be more dominated by strains of *Prevotella* compared to *Bacteroides* in more Westernized populations (19–21). Similarly, other species that are typically rarer or even undetected in Westernized populations, such as *Collinsella, Megamonas*, and *Dialister* species, appear at a higher frequency in non-Westernized populations (21, 22). A caveat to these observations is that our databases are still highly biased toward Westernized populations (18, 21, 23). Until more comprehensive representation from global populations can be achieved, the definition of a “healthy” microbiome will continue to shift, which can complicate identifying universal links to disease.

Cultivation-based discovery of the microbiota has slightly lagged behind sequencing-based definitions of a healthy microbiome. The availability of isolated species serves many purposes, including the ability to conduct mechanistic work with tangible species, further define strain-level diversity, and provide improved references for culture-independent databases. During the early days of the HMP, Fodor et al. compared HMP-generated OTUs to available whole genomes of isolated species, identifying a 1,469 “most wanted” list of OTUs that lacked cultivated representatives (24). At the time, many species present in sequence-based comparisons of the human microbiota referred to species without isolate matches as “uncultivable.” However, several large-scale cultivation efforts have been able to capture up to 100% of representative strains within the phyla encompassing the gut microbiota (25–30). Although MAG databases still lack cultivable representatives (16), most species considered prevalent across the human population have at least one representative genome that has been derived from a cultivated strain (26, 31). The availability of cultivars has enabled researchers to form synthetic communities (30, 32–34) that better represent the comprehensive ecology of a healthy microbiota, which, unlike infections caused by single pathogens, is a community effort (35). These communities have been used for mechanistic investigation of the role of the microbiota in disease in controlled settings, such as identifying factors of colonization resistance against pathogens (34) or identifying conditions and interactions that drive the production of a particular metabolite (36).

In addition to expanding species-level diversity of the human microbiota, both metagenome- and cultivation-based studies have revealed extensive intra-species variability, or strain diversity, that may further expand the functional diversity of a healthy microbiota (37–40). Pangenomic comparisons, which attempt to define all the genes carried by a particular species, have been useful for identifying functional variability across a species, as well as refining the definition of what a bacterial species actually is (41). Although pangenomics have been applied to identify genes that may distinguish a pathogen from a commensal (42), pangenomic comparisons for commensal bacterial species remain less common. For highly abundant species, pangenomic analyses have revealed additional functional diversity that further define the healthy microbiome. For instance, genomic analyses for *Bacteroides* species have uncovered high levels of genomic diversity and variability in carbohydrate-active enzymes, demonstrating variable abilities of *Bacteroides thetaiotaomicron* strains to metabolize complex polysaccharides (43–45), as well as single strain domination of an individual host by *Bacteroides fragilis* (46). Although pangenomic analyses for rarer but prevalent species remain less characterized (27), increasing database representation has allowed for some intra-species and population structure comparisons, such as within *Eubacterium rectale* (47), *Akkermansia muciniphila* (48, 49), *Clostridium innocuum* (50), *Ruminococcus gnavus* (51), and *Roseburia* species (52). A recent study published by Chen-Liaw et al. used a combination of sequencing and cultivation approaches to enumerate the strain richness across 92 prevalent gut species within most representative phyla found in an individual (45). From these data, the authors concluded that strain richness (i.e., the number of unique strains per defined species) of gut microbial species within a single individual varied from 1.0 to 2.57, suggesting lower levels of strain richness in the gut ecosystem than observed for environmental biomes such as lakes or soil, for which the calculated strain richness using the same methodology ranged from a mean of 4.93–6.83. Notably, this study also included nine species within the phylum Bacillota (Firmicutes), for which pangenomic information for many species remains under-characterized.

A final source of variability that can obscure our definition of a healthy microbiome can occur at the individual gene level. The above studies have greatly expanded our definitions of species- and strain-level variation, but mostly rely on presence vs absence of genes, whether this is applied toward core genes that define a species or accessory species that define a pangenome. Variability within individual functional gene groups, which is not always captured by set genome distances separating species or strains, can also influence our definition of a healthy microbiome. A beautiful example of how variability within even a single enzyme can influence microbiota interactions is exemplified by diverging substrate specificity of *Lactobacillus* bile salt hydrolase, the enzyme responsible for the initial step of bile salt transformation (53, 54). Recently, Foley et al. demonstrated that not only can substrate specificity for glycine- vs taurine-deconjugation shape the microbial composition in the gut (55), but also shape the outcome of *C. difficile* susceptibility (56). Combined with recent evidence that bile acids can be conjugated by other amino acids (57), reliance on mere presence/absence of a gene within a species, or even within a metagenome, may not be a sufficient measure of microbiome health.

Our ability to refine the definition of a healthy microbiome has greatly surpassed initial estimates of diversity initiated by the HMP. Yet, this progress, aided by parallel technological advances, seems to only beget more infinite diversity of the human microbiome. You might ask yourself: why does species or strain diversity even matter? I (and others) argue that defining diversity within species defines critical units needed to move the translation of the microbiome field forward. Other reviews have more comprehensively defined why strain diversity is important (39, 58, 59), which I will summarize here from my own perspective. First, further defining species-level differences continues to reveal considerable variability across human microbiomes. This suggests that we have not reached a comprehensive, or panmetagenomic, definition of a healthy microbiome. Both increasing global representation of microbial species (uncultivated and cultivated) and consideration for strain-level variance across hosts could increase our ability to design appropriate LBPs, which are not necessarily one-size-fits-all. Our ability to more precisely quantify microbial diversity of the human gut has also revealed consistent associations with certain bacterial groups that remain under-characterized, such as the class Clostridia, family Lachnospiraceae, which are commonly associated with health across human studies (60, 61). This group of bacteria includes butyrate-producers (62, 63), bile-acid transformers (54), and many spore-formers (25). Because of their correlation to health and these functional capabilities, they are often the target of many microbiome-based therapeutics (1, 61). Efforts to understand the role of these bacteria, including their strain diversity, would also greatly improve the microbiome-based design and likely represent more appropriately designed bacteria to inhabit the gut. Second, if strain-level differences matter for pathogens (64, 65), why would this not matter for commensals? Many correlative microbiota studies have relied on differences within the 16S rRNA gene, which is not usually sufficient to resolve disease associations. Indeed, studies incorporating more refined strain-level comparisons suggest that specific strains of prevalent commensals may selectively associate with certain disease states, as observed for *A. muciniphila* (49, 66) or *R. gnavus* (51, 67) strains. Beyond disease associations, strain-level variability can also influence host health via the provision of differential metabolic capacity (68) or immune induction (69). Collectively, both defining this variability and redefining the units we consider within a healthy microbiome (i.e., beyond just taxonomy) are needed to progress in the microbiome field.

## ALIGNING RECIPIENT-DONOR INTERACTIONS TO IMPROVE RECOVERY OR SUPPLEMENTATION OF MICROBIOME FUNCTIONS

Much of microbial manipulation is centered around the concept that you input a “beneficial” microbe or microbial community that then goes on to provide the desired target function or clinical outcome. This strategy has been successfully implemented with FMT to treat CDI, whereby a “healthy” microbiota is transplanted into a recipient to restore microbial functions that enact colonization resistance against *C. difficile* (70). The success of using FMT for CDI has prompted the development of recouping a healthy microbiota for other disease states, both gastrointestinal (71) and otherwise (72). This approach is also the basis for probiotic manipulation of the microbiota, whereby supplementing “good” bacteria into the gut could yield beneficial functions, even targeting specific functions such as using a butyrate-producing bacterium to decrease inflammation (73).

Logically, the first step of this process is transplanting the microbes themselves into the gut, sometimes referred to as engraftment. For FMT-based approaches to treat CDI, there is ample evidence that overall diversity, bacterial taxa associated with health, and specific functions associated with *C. difficile* resistance recover following FMT (74, 75). For mechanisms that rely on transplanting a generally diverse microbiota community to supplement or replace a perturbed microbiota, there is evidence to suggest that engraftment is correlated with clinical success. A recent study by Aggarwala et al. used metagenome- and cultivation-based approaches to determine FMT engraftment in thirteen patients with CDI, suggesting that a >17% engraftment rate could explain successful MT outcome (76). While other studies have demonstrated similar results in patients with CDI (77, 78), the engraftment rate itself may only need to be partial (79, 80). That said, several factors have been suggested to increase engraftment rate, including antibiotic use prior to FMT (78, 81), route of administration (78), or the specific microbiota profile of the recipient (82).

There is also evidence that the types of bacterial taxa that engraft from FMT-based approaches might be more important, particularly for conditions beyond CDI. Although most gut taxonomic representatives have been demonstrated to engraft from FMT products, species belonging to the Bacteroidota (i.e., *Bacteroides*, *Alistipes,* and *Prevotella* species) and Actinomycetota (*Bifidobacterium* species; *Eggerthella lenta*) phyla have been demonstrated to engraft more consistently in recipients compared to species within the Bacillota (Firmicutes) phylum (76, 78). A recent study by Schmidt et al., using MAG-based analyses to investigate FMT engraftment across multiple disease states, demonstrated high variability in both engraftment rates and strains likely to engraft (83). In the included rCDI or UC patient populations, donor strains were more likely to explain much of the post-FMT recipient composition compared to recipients with metabolic syndrome, who exhibited higher engraftment variability as well as co-existence of both donor and recipient strains. Importantly, LASSO models incorporating the recipient microbiota were larger predictors of donor engraftment, with some recipient taxa identified as “inhibitors” (*Bacteroides vulgatus* and *Bacteroides uniformis*) and others as “facilitators” (*Lactococcus lactis* and *Streptococcus salivaris*). Indeed, other studies have suggested that the extant microbiota in recipients may drive not only engraftment but clinical outcome following FMT, which is defined as successful if patient recovery or remission is observed (82, 84, 85). Promoting colonization by donated or inoculated species may be even more challenging if the goal is to transplant a specific group of bacteria or singular species. This might be particularly true for probiotic strains, many of which demonstrate variable long-term colonization in the gut (86–88).

Beyond engraftment or colonization in the gut, the supplemented microbial community or strain must also be able to provide the targeted benefit or function in the recipient microbiome. This can be complicated for multiple reasons. First, many bacterial functions involve interactions with other bacteria, once again emphasizing the importance of the extant microbiota. For instance, supplementing butyrate-producing bacteria (89) or even prebiotic compounds to support existing butyrate-producing bacteria in the gut (90) does not automatically translate into increased butyrate, as overall butyrate yield (either via production or consumption) is modulated by interactions with other bacteria (36, 63). Second, there is evidence to suggest that beyond selective host or environmental barriers to colonization, interactions with the host may influence how engrafted microbes function in the recipient environment. This has been observed in mouse models of CDI, whereby FMT was not successful in clearing *C. difficile* in mice with impaired CD4 T cells (91) or human-sourced FMT was unable to clear *C. difficile* in mice harboring their indigenous microbiome (92). In both studies, engraftment and colonization by “beneficial” taxa were observed, suggesting that both host and microbial features of the recipient gut play a role in determining the impact of microbial manipulation.

Perhaps more importantly, the exact functions or mechanisms to target most conditions remain obscure. Even for successful FMT-based treatment of CDI, identification of the exact mechanisms to clear *C. difficile* (other than replacing the recipient community with a rich donor microbiota) has likely hampered the development of successful synthetic communities. For probiotic microbial manipulation, the search for mechanisms is even more unclear. For example, while probiotic use for conditions ranging from idiopathic diarrhea to neonatal sepsis can be mechanistically dissected *in vitro* (93) or using animal models (94), validation of mechanism is difficult to study in humans. Both systematic reviews and large-scale clinical trials in humans have demonstrated opposing conclusions for many conditions (95). Inconsistency in analyses is to be expected when clinical outcome or self-reported benefit represents the only measurable outcome. Relatedly, the use of probiotics to generally aid recovery of a “healthy” microbiota can also have unspecified or undesirable effects. While the most commonly used probiotic species (*Lactobacillus, Bifidobacterium, Streptococcus*, or *Enterococcus* species) are indeed gut inhabitants (96), the actual commercial strains may be far-removed from the species originally isolated from the human gut. Using paired human and mouse studies, Suez et al. demonstrated how supplementation with an 11 species probiotic community (consisting of commonly available probiotic strains) in antibiotic-treated mice or humans attenuated recovery of the microbiota to baseline levels (88). While the study did not investigate how this altered microbiota trajectory influenced host health or disease susceptibility, the effect of using non-specific or probiotic strains less relevant to the gut adds to the ambiguity surrounding probiotic benefits.

Nevertheless, several approaches have been proposed to overcome the engraftment and functional barriers involved in microbial manipulation. The use of strains or species more closely resembling those associated with health might represent more effective probiotic or pharmaceutical approaches, previously referred to as “next-generation” probiotics (97). Many of these species fall under the aforementioned Bacillota (Firmicutes) phylum, most of which are highly intolerant to oxygen. To overcome this preparatory barrier, Khan et al. adapted a strain of the butyrate-producing bacterium *Faecalibacterium prausnitzii* to be more aerotolerant, increasing the viability of the strain for probiotic use in humans (98). The authors additionally co-cultured and co-administered *F. prausnitzii* with *Desulfovibrio piger*, a sulfate-reducing bacterium that could potentially complement or even increase the butyrate-producing capacity of *F. prausnitzii*. Similar cross-feeding approaches, using either co-culture with another bacterium or a substrate (i.e., “prebiotic”), have also demonstrated increased colonization (99) or functional capacity (100). Finally, genetic engineering of bacteria to better colonize or constitutively produce a target function represents an exciting avenue for live biotherapeutic delivery of functions in the gut, either via adding and/or deleting target genes, or engineering constitutive or inducible promoters to express the desired target gene independent of environmental drivers (101). While this approach has been mostly limited to genetically tractable organisms such as *Escherichia coli* or *B. theta* (102–104), advances in genetic manipulation will hopefully aid progress of genetically engineered nonmodel organisms, including many Bacillota members within the gut, for both experimental and therapeutic purposes (105–107).

A final consideration is that microbial manipulation, whether via the use of LBPs to target specific conditions or probiotics to improve general wellness, represents an ecological problem (108, 109). The above collective data suggests that beyond identifying appropriate functional targets for microbial manipulation, interactions with both extant microbes and host features are driving forces for the establishment and impact of newly introduced microbes. A recent commentary emphasized the need for more classical microbiology in microbiome research (110); I could not agree more, particularly for identifying the mechanisms to target during disease development or prevention. But we cannot forget the ecological forces that ultimately determine how individual microbes colonize, persist, evolve, and interact with each other, all of which ultimately influence community-level functions and the host. This reverberates throughout defining strain-level differences across healthy microbiomes as well as achieving the desired impact with microbial manipulation.

## CONCLUSION

While the above considerations might seem to emphasize barriers to developing more effective means of manipulating our microbiomes for health, these contexts are only possible for consideration due to the steadfast progress in the microbiome field, particularly within the last two decades. Discoveries and developments surrounding the microbiome have only been possible due to continued funding of basic and translational research of interdisciplinary teams of science, including microbiologists, ecologists, bioinformaticians, immunologists, and clinicians. Since the launch of the HMP (which coincides with when I began my graduate studies and first learned about the microbiome), it has been amazing to watch this progress in real time. I look forward to another decade of progress in the development of microbiome-based therapeutics, with additional discoveries and new considerations that I cannot even imagine today.
